# Knowledge of Primary Health Care Physicians About the Novel Class of Type 2 Diabetes Medication in Qassim Province, Saudi Arabia

**DOI:** 10.7759/cureus.86638

**Published:** 2025-06-24

**Authors:** Renad Alghofaili, Chandra Sekhar

**Affiliations:** 1 Family Medicine, Family Medicine Academy, Qassim Health Cluster, Buraidah, SAU

**Keywords:** dpp-4 inhibitors, glp-1 ra, knowledge, phcc physicians, qassim province, saudi arabia, sglt-2 inhibitors

## Abstract

Background: Type 2 diabetes mellitus (T2DM) is a worldwide issue, stemming from multiple factors, primarily environmental and lifestyle habits. New medications are constantly emerging due to the chronic nature of the disease and its impact on various organ systems. The competitive market requires primary health care center (PHCC) physicians to use accurate doses, treatment durations, and novel medication practices to benefit their patients. This study aims to assess Qassim PHCC physicians' knowledge of a new class of T2DM medication.

Methods: A cross-sectional study was conducted among 230 PHCC physicians using a semi-structured self-administered questionnaire, and 191 responded. A simple random method was applied to select 11 governorates from Qassim province. Then, the self-administered questionnaire link was distributed through WhatsApp, and direct messages were sent in a convenient manner to each governorate supervisor and PHCC physician. Data analysis was performed using Statistical Program for Social Sciences (SPSS) version 21 (IBM Corp., Armonk, NY), and the chi-square test was applied for categorical analysis.

Results: The study revealed that the mean age of Qassim PHCC physicians was 33.66±7.6 years, with 72.3% aged 25-35 years. Males comprised 62.3% of the group, and professional classifications included 22.5% general physicians (GPs), 33.5% family medicine (FM) residents, 28.3% FM specialists, and 15.7% FM consultants. While 88% encountered type 2 diabetes daily, only 36.1% received continued medical education (CME) training on novel medications. Awareness of glucagon-like peptide-1 receptor agonists (GLP-1 RAs) (94.8%), dipeptidyl peptidase-4 inhibitors (DPP-4is) (83.8%), and sodium-glucose co-transporter-2 inhibitors (SGLT-2is) (86.9%) was high, yet American Diabetes Association (ADA) guideline familiarity varied, significantly higher among FM specialists and consultants. GLP-1 RAs were linked to the greatest glycated hemoglobin (HbA1c) reduction (2.07% ± 0.92), while SGLT-2i and DPP-4i followed with 1.53% ± 0.78 and 1.25% ± 1.01 reductions, respectively. Most physicians (92.7%) recognize the cardiovascular benefits of GLP-1 RAs, but educational needs are required. There are significant awareness disparities between professional roles (FM specialist and FM consultant versus GP and FM residents) and with all three novel medications (p<0.05).

Conclusions: The study highlights significant gaps in CME among Qassim PHCC physicians, with only 36.1% trained in novel diabetes medications despite daily encounters with patients with T2DM. While awareness of GLP-1 RAs and other medications was high, variations in ADA guideline familiarity indicate a need for role-specific educational interventions. Physicians acknowledged GLP-1 RAs' superior HbA1c reduction and cardiovascular benefits. Policymakers should prioritize targeted CME programs, enhance access to updated guidelines, and promote tailored training to bridge knowledge and practice gaps.

## Introduction

Diabetes mellitus (DM), a metabolic condition known as chronic hyperglycemia, is characterized by either insufficient insulin secretion, ineffective insulin, or both [1]. It is mainly classified into many types, the most prevalent one is type 2, accounting for 90% of cases [2]. It is one of the major health issues of the 21st century, a chronic global epidemic, and one of the fastest-growing global health concerns. Around 537 million individuals were estimated to have diabetes in 2021; by 2030, that figure is expected to rise to 643 million, and by 2045, it is predicted to reach 783 million. Furthermore, it was projected that 541 million individuals would have impaired glucose tolerance in 2021 [2]. With an estimated 7 million people living with diabetes and over 3 million with pre-diabetes, Saudi Arabia has the second-highest rate of diabetes in the Middle East and the seventh-highest rate worldwide, according to the WHO [3]. Without a decisive, comprehensive intervention effort, the economic burden of DM in Saudi Arabia is poised to become catastrophic [4].

A multidisciplinary strategy is required to control the prevalence, including patient education and healthcare practitioners' knowledge updates [5]. In addition to glycemic control, which lessens the consequences of persistent hyperglycemia, treatment for DM is multifactorial and complex [6]. Several classes of antidiabetic drugs, such as sulphonylureas, biguanides, meglitinides, thiazolidinediones, and alpha-glucosidase inhibitors, are available for treating DM. Although these medications are extensively used, optimum achievement in the management of the disease is a far-reaching goal and sometimes needs second-line drugs for adequate control of hyperglycemic situations [7].

These medications are associated with potential adverse effects, which make the treatment a challenging task. Studies have suggested that due to complications and unwanted side effects, patients themselves stop the medications, resulting in several long-term complications of mismanaged hyperglycemia [8]. According to recent research, the incretin system may be a key target for treating type 2 DM (T2DM). The gut mucosa produces incretins, which are hormones, in response to meals. Glucagon-like peptide-1 (GLP-1) and glucose-dependent insulinotropic polypeptide (GIP) are the two identified incretin hormones [9]. A more recent family of drugs known as GLP-1 receptor agonists (RAs) has been approved to treat adult T2DM. It is well known that medications lower blood glucose levels to a considerable degree [10].

According to the drug's safety profile, using GLP-1 RA can raise the risk of thyroid C-cell neoplasia, cause pancreatitis, and cause local irritation and stomach distress. The medication is contraindicated in pregnancy and lactating mothers, as the safety profile in these patients is incomplete [11]. Previous research has produced conflicting information regarding people's knowledge, attitudes, and practices (KAP) regarding several drugs. It has been documented that inadequate KAP might make treatment plans more difficult to implement while managing long-term conditions such as DM [12].

A study carried out in India revealed that patients exhibited a much lower level of awareness regarding the rationale behind the use of insulin in the treatment of DM [13]. One approach is to periodically measure medical professionals' KAP through scientific and statistical surveys. The results can help develop better methods for exchanging medical knowledge among practitioners [14].

Primary health care center (PHCC) physicians, as first points of contact, play a vital role in managing diabetes [15]. The research from Saudi Arabia highlights the factors that influence their decision to prescribe GLP-1 RAs [16]. Studies show variations in knowledge and usage globally, with gaps in understanding among physicians in the United Kingdom [17], and the need for education on complex insulin regimens, observed in a 2015 study [18]. The study emphasized the importance of managing T2DM effectively, clarified the differences between dipeptidyl peptidase-4 inhibitors (DPP-4is) and GLP-1 RAs, and outlined strategies for advancing their use in diabetes treatment [18]. In Saudi Arabia, healthcare professionals show good understanding but need updates on the side effects of novel T2DM medication [19].

The study concluded that awareness about the optimum use of GLP-1 RA medication is essential for achieving better glycemic control [19]. However, it did not discuss the other two novel classes of medications, DPP-4i and sodium-glucose co-transporter 2 (SGLT-2i), and assess the knowledge of PHCC physicians. This highlights the need to conduct the study in our PHCC of Qassim to bridge the research gap. T2DM is one of the significant public health problems throughout the world, including in Saudi Arabia. Moreover, this study included KAP among Qassim University healthcare professionals, not among PHCC physicians [19].

GLP-1 RA drugs have shown promising potential in managing T2DM and improving patient outcomes [10]. However, underutilization of GLP-1 RA and SGLT-2i drugs in the Riyadh study underscores the need for better awareness and education for optimal diabetes care [20]. Family physicians' knowledge and prescribing patterns regarding these medications in the Qassim region have not been extensively studied. This research aims to evaluate awareness, perceptions, and prescription practices about a novel class of T2DM medication among PHCC physicians. These results will shed light on the optimum benefit of implementing glycemic control strategies in the future for T2DM patients through policymakers.

Objective 

This study aimed to determine the physicians' knowledge and demographic associations regarding GLP-1 RA, DPP-4i, and SGLT-2i medication for the management of T2DM, focusing on their understanding of indications, contraindications, complications, and patient selection criteria.

## Materials and methods

Study setting and target population

Qassim province had 12 governorates, and the capital city of Qassim province is Buraidah city [21]. We involved 11 governorates from the Qassim province (including Buraidah, Unaizah, Al Rass, Albadaya, Albukharya, Al Mithnab, Al Nabhaniyah, Riyadh Al Khabra, Uqlat al-Ṣuqur, Uyun AlJiwa, and Dariyah) to select the study setting and the PHCC of Qassim. From each governorate, PHCC physicians, such as general physicians (GPs), family medicine (FM) residents, and family physicians (specialists and consultants), who worked in the above-mentioned cities and rural areas of PHCC were included.

Study design

A cross-sectional study was designed and applied among the PHC physicians during the period from November 2024 to February 2025.

Sample size and sampling method 

Based on the OpenEpi sample size calculator application [22], as per the Qassim Health Cluster information, the total PHCC physician population in Qassim province was 563 [23]. The anticipated prevalence was 50%, with a 95% CI and design effect of 1. The estimated sample was 230.

The study included the cities and rural areas of Qassim province, as mentioned above. The governorates were selected by using a simple random method, and study participants were selected by using a convenience sampling method. According to the Ministry of Health Statistics, the Qassim province had 153 PHCC [23]. Initially, the principal investigator met the concerned governorate's supervisors and discussed about reaching the PHC physicians. Then, the concerned governorates' supervisor advocated for sharing the physicians' contact numbers. Then, the principal investigator distributed the self-administered semi-structured questionnaire through social media via Google Forms, with frequent reminders once a week. 

Inclusion and exclusion criteria

PHCC physicians and FM residents belong to Qassim province and are of both genders. Other physicians working at the polyclinics, private hospitals, and other governmental hospitals, as well as non-cooperative participants, were excluded. 

Data collection tool and procedure

We reviewed literature through the PUBMED and Google Scholar search engines based on our initial research idea. We found some studies and reviewed them, and based on the support of the literature, we constructed a questionnaire. The majority of knowledge questions about GLP-1 RA were taken from a local Qassim study published in the year 2021 and were also communicated by mail to the corresponding author, and this article is available in open access mode, including a questionnaire [19].

The questionnaire consists mainly of five sections. The first section includes demographic characteristics such as age, gender, years of experience, and position of physicians. The second section comprises 13 knowledge-based questions on GLP-1 RA. The third section consists of five knowledge questions on DPP-4i, while the fourth section contains five questions on SGLT-2i. The fifth section of the questionnaire covers certain variables, such as the frequency of encounters with patients with DM in their clinics, training status/continued medical education (CME) on a novel class of medication, awareness of American Diabetes Association (ADA) guidelines, physicians' preference about medication, and the reduction of glycated hemoglobin (HbA1c) level after initiation of the new class of DM medication in 6 months. After the questionnaire was developed and new variables were included, it was reviewed by the FM consultants and research-experienced faculty for their input and approval to use for the validation process. 

Pilot study 

A pilot study was conducted in the field among 10 physicians to determine the technical feasibility and validity of the questionnaire to ensure a good presentation and order of the questions. The pilot study sample was not included in the main study sample. After the pilot study, we did not change our questionnaire.

Ethical considerations

The data were collected after obtaining approval from the Regional Ethics Committees, with an approval number 607-45-15565 dated May 29, 2024. The permission of the concerned selected governorate supervisor was obtained before the data collection procedure. Informed consent was inferred through the questionnaire's first page, which was obtained from every participant, and confidentiality of the information was maintained. Data were not shared with any public or private agencies.

Statistical analysis

The data were entered, cleaned, and analyzed by the Statistical Package for Social Sciences (SPSS) version 21 (IBM Corp., Armonk, NY). Demographic characteristics were mentioned in frequencies and percentages. Means and standard deviations were calculated for the study's continuous variables of age and HbA1c levels. For all the categorical variables, such as age, gender, and professional classification with GLP-1 RA, DPP-4i, and SGLT-2i medication knowledge, the chi-square test was applied. The level of statistical significance was set at the CI of 95%, as the p-value was ≤0.05. 

## Results

In the present study, about 191 physicians participated and distributed a questionnaire to approximately 230 physicians. The response rate in the study population was 83% (191/230).

Table 1 shows that the mean age and standard deviation of Qassim PHCC physicians in the study population were 33.66±7.6 years, and the mean years of experience of PHCC physicians in the study group were 6.84±6.29 years. About 72.3% (n=138) of PHCC physicians aged 25-35 years were in the study population. In the study group, 62.3% (n=119) were males. Among the professional classification of PHCC physicians, 22.5% (n=43) were GPs, 33.5% (n=64) were FM residents, 28.3% (n=54) were FM specialists, and 15.7% (n=30) were FM consultants. Nearly 88% (n=168) of physicians mentioned the daily encounters of patients with type 2 diabetes.

**Table 1:** Demographic characteristics of the physicians working at the PHCC of Qassim FM, family medicine; GP, general practitioner; PHCC, primary health care center; SD, standard deviation

Demographic variables	Number of participants	Percentage
Age ± SD	33.66±7.6 years	
Years of experience ± SD	6.84±6.29 years	
Age category		
25-35 years	138	72.3
36-45 years	32	16.8
>45 years	21	11.0
Gender		
Male	119	62.3
Female	72	37.7
Professional position		
GP	43	22.5
FM resident	64	33.5
FM specialist	54	28.3
FM consultant	30	15.7
Frequency of encounters of patients with type 2 diabetes		
Daily	168	88.0
Once in 2 days	17	8.9
Weekly once	6	3.1

Table 2 shows that only 36.1% (n=69) of physicians reported receiving CME training on novel diabetes medications, while 63.9% (n=122) reported not receiving such training. Around 82.2% (n=157) of physicians had access to educational resources related to GLP-1 RAs, 85.9% (n=164) to DPP-4is, and 85.3% (n=163) to SGLT-2is. A high proportion of physicians were aware of ADA recommendations regarding GLP-1 RAs (86.4%, n=165), DPP-4is (83.8%, n=160), and SGLT-2is (86.9%, n=166). According to the latest ADA guidelines, 62.3% (n=119) of physicians favor GLP-1 RAs as the preferred choice when a first-line treatment fails, while 37.7% (n=72) opt to use them at any point in treatment. Similarly, 58.1% (n=111) of physicians preferred DPP-4is when a first-line option fails, and 41.9% (n=80) would use them anytime. For SGLT-2is, 61.3% (n=117) of physicians selected them when a first-line option fails, while 38.7% (n=74) would use them at any time.

**Table 2:** Status of physicians' CME training, educational resources, awareness about ADA guidelines, and novel classes of medication preference in the study population ADA, American Diabetes Association; CME, continuing medical education; DPP-4i, dipeptidyl peptidase-4 inhibitor; GLP-1 RA, glucagon-like peptide-1 receptor agonist; SGLT-2i, sodium-glucose co-transporter 2 inhibitor

Variables	No (%)	Yes (%)	Total (%)
CME on novel DM medication	122 (63.9)	69 (36.1)	191 (100)
Educational resources			
GLP-1 RA	34 (17.8)	157 (82.2)	191 (100)
DPP-4i	27 (14.1)	164 (85.9)	191 (100%
SGLT-2i	28 (14.7)	163 (85.3)	191 (100)
Physicians knowledge			
ADA awareness: GLP-1 RA	26 (13.6)	165 (86.4)	191 (100)
ADA awareness: DPP-4i	31 (16.2)	160 (83.8)	191 (100)
ADA awareness: SGLT-2i	25 (13.1)	166 (86.9)	191 (100)
Awareness of the latest ADA guidelines and preference	First-line drugs fail (%)	Any time (%)	Total (%)
Latest ADA: GLP-1 RA	119 (62.3)	72 (37.7)	191 (100)
Latest ADA: DPP-4i	111 (58.1)	80 (41.9)	191 (100)
Latest ADA: SGLT-2i	117 (61.3)	74 (38.7)	191 (100)

Table 3 depicts physicians' perceptions about the GLP-1 RA, which showed the highest mean HbA1c reduction at 2.07% ± 0.92 after six months of treatment. SGLT-2i had a mean HbA1c reduction of 1.53% ± 0.78. DPP-4i had the lowest reduction at 1.25% ± 1.01. These findings highlight physicians’ awareness and preferences regarding novel diabetes medications and their expectations of HbA1c reduction efficacy.

**Table 3:** Physicians' perceptions on the experience of reducing HbA1c levels with new diabetes medication over six months of treatment DPP-4i, dipeptidyl peptidase-4 inhibitor; GLP-1 RA, glucagon-like peptide-1 receptor agonist; HbA1c, glycated hemoglobin; SD, standard deviation; SGLT-2i, sodium-glucose co-transporter 2 inhibitor

Variables of a new class of diabetes medication	Perceptions of mean HbA1c percentage reduction ± SD
Mean reduction of HbA1c with 6 months of treatment with GLP-1 class of drugs ± SD	2.07% ± 0.92
Mean reduction of HbA1c with 6 months of treatment with DPP-4 class of drugs ± SD	1.25% ± 1.01
Mean reduction of HbA1c with 6 months of treatment with SGLT-2i class of drugs ± SD	1.53% ± 0.78

Table 4 presents the knowledge of PHCC physicians regarding GLP-1 RAs. The majority, 94.8% (n=181), of physicians were aware of GLP-1 RAs, and 86.9% (n=166) recognized them as the latest addition to T2DM management. Most respondents, 83.8% (n=160), knew that GLP-1 RA does not cause hypoglycemia, and 81.7% (n=156) acknowledged its role in lowering blood pressure. Awareness of potential side effects, such as pancreatitis (89%, n=170) and thyroid neoplasm risk (84.8%, n=162), was also high. However, only 79.1% (n=151) of physicians were familiar with the latest ADA 2024 recommendations regarding GLP-1 RA. Knowledge about contraindications, such as severe renal impairment (84.3%, n=161), pregnancy and breastfeeding (91.1%, n=174), and gastroparesis (83.8%, n=160), was also assessed. Additionally, 93.7% (n=179) of physicians were aware of the different routes of administration, while 92.7% (n=177) understood its cardiovascular benefits.

**Table 4:** PHCC physicians' knowledge of GLP-1 RA medication ADA, American Diabetes Association; GLP-1 RA, glucagon-like peptide-1 receptor agonist; PHCC, primary health care center

Knowledge questions about GLP-1 RA	No (%)	Yes (%)
Do you know GLP-1 RA?	10 (5.2)	181 (94.8)
Do you know that GLP-1 RA is the latest addition in the management of type 2 diabetes mellitus?	25 (13.1)	166 (86.9)
Is it true that administration of GLP-1 RA does not cause hypoglycemia in patients with type 2 diabetes mellitus?	31 (16.2)	160 (83.8)
Is it true that the use of GLP-1 RA can cause a reduction in blood pressure?	35 (18.3)	156 (81.7)
Are you aware that GLP-1 RA therapy can cause pancreatitis in patients WITH diabetes?	21 (11.0)	170 (89.0)
Do you agree that GLP-1 RA carries a potential risk of thyroid C-cell neoplasm?	29 (15.2)	162 (84.8)
Are you aware of ADA recommendations in 2024 in regard to GLP-1 RA?	40 (20.9)	151 (79.1)
Do you agree that severe renal impairment is considered a contraindication for GLP-1 RA?	30 (15.7)	161 (84.3)
Do you agree that pregnancy and breastfeeding are considered as a contraindication for GLP-1 RA?	17 (8.9)	174 (91.1)
Do you agree that gastroparesis is considered a contraindication for GLP-1 RA?	31 (16.2)	160 (83.8)
Are you aware of different doses of GLP-1 RA?	25 (13.1)	166 (86.9)
Are you aware of GLP1 drug routes of administration?	12 (6.3)	179 (93.7)
Do you understand the role of GLP-1 RA in reducing cardiovascular risk factors in patients with diabetes?	14 (7.3)	177 (92.7)

Table 5 shows that, regarding the awareness of ADA recommendations for GLP-1 RA, 76.8% (n=106) of PHCC physicians were aware in the age group of 25-35 years, 87.5% (n=28) were aware in the age group of 36-45 years, and 81% (n=17) were aware over 45 years. No statistically significant association was observed between the different age groups compared to ADA recommendations of GLP-1 RA (p=0.398). About 80.7% (n=96) of male physicians were aware of ADA recommendations of GLP-1 RA, whereas only 76.4% (n=55) of female physicians were aware. No significant association was observed between gender and ADA recommendations of GLP-1 RA (p=0.481). Regarding the professional position of PHC physicians, among GPs and FM residents, only 68.2% (n=73) were aware of ADA recommendations for GLP-1 RA. In contrast, 92.9% (n=78) of FM specialists and consultants were aware of ADA recommendations of GLP-I RA, and this association was statistically significant (p=0.0001). Concerning the frequency of patient encounters with T2DM, 81% (n=136) of physicians were aware of ADA recommendations among daily visitors, 64.7% (n=11) were aware in once in two-day visits, and 66.7% (n=4) were aware in once-weekly visits (p=0.219).

**Table 5:** Sociodemographic variables associated with PHCC physicians' knowledge of ADA recommendations for GLP-1 RA medication ¶: Significant p-value ADA, American Diabetes Association; FM, family medicine; GP, general practitioner; PHCC, primary health care center; p, probability; T2DM, type 2 diabetes mellitus; X², chi-square

Sociodemographic variables	No awareness of ADA recommendations for GLP-1 RA	Awareness of ADA recommendations for GLP-1 RA	X^2^ value	p-value
Age categories				
25-35 years	32 (23.2%)	106 (76.8%)	1.844	0.398
36-45 years	4 (12.5%)	28 (87.5%)		
>45 years	4 (19.0%)	17 (81.0%)		
Gender				
Male	23 (19.3%)	96 (80.7%)	0.497	0.481
Female	17 (23.6%)	55 (76.4%)		
Professional position				
GP + FM resident	34 (31.8%)	73 (68.2%)	17.246	0.0001^¶^
FM specialist + FM consultant	6 (7.1%)	78 (92.9%)		
Frequency of patients encounter with T2DM				
Daily	32 (19.0%)	136 (81.0%)	3.036	0.219
Once in 2 days	6 (35.3%)	11 (64.7%)		
Once weekly	2 (33.3%)	4 (66.7%)		

Table 6 assesses the knowledge of physicians regarding DPP-4i. A high percentage, 84.8% (n=162), of physicians were aware of the different doses, and 92.7% (n=177) knew the route of administration. About 85.3% (n=163) of physicians reported using DPP-4is alone or in combination with other antidiabetic medications, such as SGLT-2is. Regarding contraindications, 89% (n=170) of physicians agreed that pregnancy and breastfeeding are contraindications for DPP-4is. However, knowledge about stopping DPP-4is 90 days before conception was lower, with 77.5% (n=148) of physicians correctly identifying this recommendation.

**Table 6:** PHCC physicians' knowledge questions about DPP-4is DPP-4i, dipeptidyl peptidase-4 inhibitor; PHCC, primary health care center; SGLT-2i, sodium-glucose co-transporter 2 inhibitor.

Knowledge questions about DPP-4is	No (%)	Yes (%)
Are you aware of different doses of DPP-4is?	29 (15.2)	162 (84.8)
Are you aware of the route of administration of DDP-4is?	14 (7.3)	177 (92.7)
Do you use DPP-4is alone or in combination with other novel classes of diabetic medication (SGLT-2is)	28 (14.7)	163 (85.3)
Do you agree that pregnancy and breastfeeding are considered contraindications for DPP-4i?	21 (11.0)	170 (89.0)
Do you agree that 90 days before conception of pregnancy, the DPP-4is must be stopped?	43 (22.5)	148 (77.5)

Table 7 examines the association between sociodemographic variables and physicians' knowledge of DPP-4is. Knowledge about DPP-4is was highest among physicians aged 36-45 years (93.8%, n=30), while those aged 25-35 years (84.1%, n=116) and above 45 years (81.0%, n=17) had slightly lower awareness levels. However, the association between DPP-4i knowledge and physicians in different age groups was insignificant (p=0.314). Male (84.0%, n=100) and female (87.5%, n=63) physicians had close to similar knowledge levels, with no significant difference in DPP-4i knowledge (p=0.512). FM specialists and consultants had a significantly higher DPP-4i knowledge (95.2%, n=80) than GPs and FM residents (77.6%, n=80) (p=0.001). Regarding the frequency of patient encounters, physicians who saw patients daily had significantly higher DPP-4i knowledge (89.3%, n=150) compared to those seeing patients once every two days (64.7%, n=11) or once weekly (33.3%, n=2) (p=0.0001).

**Table 7:** Demographic variables associated with PHCC physicians' knowledge of DPP-4i medication use alone or in combination with other novel medication classes ¶: Significant p-value DPP-4i, dipeptidyl peptidase-4 inhibitor; FM, family medicine; GLP-1 RA, glucagon-like peptide-1 receptor agonist; GP, general physician; PHCC, primary health care center; p, probability; SGLT-2i, sodium-glucose co-transporter 2 inhibitor; T2DM, type 2 diabetes mellitus; X², chi-square

Sociodemographic variables	No knowledge of DPP-4i use alone or in combination with other novel classes of diabetic medication	Knowledge of DPP-4i use alone or in combination with other novel classes of diabetic medication	X^2^ value	p-value
Age categories				
25-35 years	22 (15.9%)	116 (84.1%)	2.314	0.314
36-45 years	2 (6.3%)	30 (93.8%)		
>45 years	4 (19.0%)	17 (81.0%)		
Gender				
Male	19 (16.0%)	100 (84.0%)	0.431	0.512
Female	9 (12.5%)	63 (87.5%)		
Professional position			11.742	0.001^¶^
GP + FM resident	24 (22.4%)	83 (77.6%)		
FM specialist + FM consultant	4 (4.8%)	80 (95.2%)		
Frequency of patients encounter with T2DM				
Daily	18 (10.7%)	150 (89.3%)	20.848	0.0001^¶^
Once in 2 days	6 (35.3%)	11 (64.7%)		
Once weekly	4 (66.7%)	2 (33.3%)		

Table 8 evaluates physicians' knowledge about SGLT-2is. Awareness of different doses was relatively high, 88% (n=168), and most physicians, 96.3% (n=184), were familiar with the route of administration. Knowledge of contraindications, including pregnancy and breastfeeding, 88.5% (n=169), was also strong. However, fewer physicians, 78% (n=149), knew the need to discontinue SGLT-2is 90 days before conception. Additionally, 75.9% (n=145) knew SGLT-2is should be stopped three days before elective surgery, indicating a gap in awareness regarding preoperative management.

**Table 8:** PHCC physicians' knowledge questions about SGLT-2is SGLT-2i, sodium-glucose co-transporter 2 inhibitor; PHCC, primary health care center

Knowledge questions about SGLT-2is	No (%)	Yes (%)
Are you aware of different doses of SGLT-2is?	23 (12.0)	168 (88.0)
Are you aware of the route of administration of SGLT-2is?	7 (3.7)	184 (96.3)
Do you agree that pregnancy and breastfeeding are considered contraindications for SGLT-2i?	22 (11.5)	169 (88.5)
Do you agree that 90 days before conception, the SGLT-2s must be stopped?	42 (22.0)	149 (78.0)
Do you know SGLT-2is are to be stopped three days before elective surgery?	46 (24.1)	145 (75.9)

Table 9 explores factors influencing physicians’ knowledge of different doses of SGLT-2is. Awareness was highest among physicians aged 36-45 years (96.9%, n=31) and those aged 25-35 years (87.0%, n=120), while those above 45 years had slightly lower awareness (81.0%, n=17). The association between the physicians' age groups and their knowledge of different doses of SGLT-2i was not statistically significant (p=0.173). Knowledge levels were comparable between male physicians (87.4%, n=104) and female physicians (88.9%, n=64), with no significant difference (p=0.759). FM specialists and consultants had significantly higher awareness about different doses of SGLT-2is (97.6%, n=82) compared to GPs and FM residents (80.4%, n=86) (p=0.0001). Concerning the frequency of patient encounters, physicians who saw patients daily had significantly higher knowledge (90.5%, n=152) compared to those seeing patients once every two days (70.6%, n=12) or once weekly (66.7%, n=4) (p=0.015).

**Table 9:** Sociodemographic variables associated with PHCC physicians' knowledge about different doses of SGLT-2i medication ¶: Significant p-value FM, family medicine; GP, general physician; PHCC, primary health care center; p, probability; SGLT-2i, sodium-glucose co-transporter 2 inhibitor; T2DM, type 2 diabetes mellitus; X², chi-square

Sociodemographic variables	Knowledge about different doses of SGLT-2i absent	Knowledge about different doses of SGLT-2i present	X^2^ value	p-value
Age categories				
25-35 years	18 (13.0%)	120 (87.0%)	3.506	0.173
36-45 years	1 (3.1%)	31 (96.9%)		
>45 years	4 (19.0%)	17 (81.0%)		
Gender				
Male	15 (12.6%)	104 (87.4%)	0.095	0.759
Female	8 (11.1%)	64 (88.9%)		
Professional position				
GP + FM resident	21 (19.6%)	86 (80.4%)	13.213	0.0001^¶^
FM specialist + FM consultant	2 (2.4%)	82 (97.6%)		
Frequency of patients encounter with T2DM				
Daily	16 (9.5%)	152 (90.5%)	8.416	0.015^¶^
Once in 2 days	5 (29.4%)	12 (70.6%)		
Once weekly	2 (33.3%)	4 (66.7%)		

## Discussion

Our study aimed to assess the knowledge of PHCC physicians in Qassim regarding novel classes of type 2 diabetes medications, including GLP-1 RA, DPP-4i, and SGLT-2i. Understanding physicians' knowledge is crucial, as it directly impacts the management of diabetes and the effectiveness of glycemic control strategies. Accurate knowledge of these medications can improve patient outcomes and reduce the complications associated with T2DM. This study provides valuable insights into the existing knowledge gaps and highlights areas requiring targeted educational interventions.

In our study, physicians demonstrated a high level of awareness regarding the effectiveness of GLP-1 RA, which led to the highest HbA1c reduction (2.07% ± 0.92) after 6 months, followed by SGLT-2i (1.53% ± 0.78) and DPP-4i with the lowest reduction (1.25% ± 1.01). Regarding HbA1c, these findings highlight physicians’ awareness and preferences regarding novel diabetes medications and their expectations of HbA1c reduction efficacy, reflecting their expectations based on clinical outcomes. A study from Japan stated that HbA1c reductions occurred within 90 days of treatment after the initiation of add-on treatments. Fasting plasma glucose and HbA1c reductions were reduced with once-weekly GLP-1 medication [24]. GLP-1 seems to be a better option for the reduction of HbA1c among patients with uncontrolled T2DM than other novel medications such as DPP-4i and SGLT-2i [24]. A study from China reported GLP1 RA to be superior to SGLT-2i in reducing HbA1c, with a mean difference of -0.39% (95% CI -0.70 to -0.08) [25]. In another systematic review study from China, Chai et al. stated that a combination of GLP-1 RA, DPP-4i, and SGLT-2i medication significantly reduces the HbA1c levels [26]. A study from the USA also stated that when it comes to the choice of novel medication for glycemic control, GLP-1 RA had an upper edge over the DPP-4i medication in the areas of HbA1c reduction, which was observed in many clinical trials [27].

Our study results indicated that most respondents (83.8%) knew that GLP-1 RA does not cause hypoglycemia, and 81.7% recognized its blood pressure-lowering effect. A study from China also stated that GLP-1 RA decreases hyperglycemia and hypertension [25]. Additionally, a high percentage of respondents were knowledgeable about side effects such as pancreatitis (89%) and thyroid neoplasm risk (84.8%). However, awareness of the latest ADA 2024 recommendations was slightly lower (79.1%), and 84.3% were aware of contraindications such as severe renal impairment, suggesting a need for continuous medical education. A study from Saudi Arabia reported similar findings, highlighting that a considerable proportion of physicians are well-informed about the benefits and risks of GLP-1 RA but require regular updates on evolving guidelines [19]. On the contrary, a study from the USA involving patients with T2DM reported that GLP-1 RAs did not increase the risk of pancreatitis, and their use was associated with a low lifetime risk of this condition [28]. This finding was supported by the European Medicines Agency and the Food and Drug Administration, which jointly conducted a comprehensive analysis of toxicology studies, revealing that they are less prone to pancreatitis following GLP-1 RA [29].

Our study found no significant association between age or gender and the knowledge of ADA recommendations for GLP-1 RA (p=0.398). However, a significant difference was based on professional positions; 92.9% of FM specialists and consultants were aware of ADA guidelines compared to 68.2% of GPs and FM residents (p=0.0001). A 2023 study in the USA among endocrinologists, primary care physicians (PCPs), and advanced practice providers (APPs) found that 65% of healthcare providers were very/extremely familiar with GLP-1 RA. The awareness was 82.5% among endocrinologists, 55.8% among PCPs, and 50.6% among APPs [30].

This suggests that experience and specialization may be critical in enhancing knowledge and clinical inertia about novel diabetes medications. A 2014 study from the United States on PCPs and their educational needs also reported similar patterns, indicating that higher specialization correlates with better awareness of updated guidelines and medication practices [18]. A study from endocrinologists and PCPs highlighted cost as a barrier to appropriate therapy for obesity, with PCPs more familiar with GLP-1 RAs than glucagon or GIP RAs. These treatments offer cardiometabolic benefits beyond weight loss, including managing non-alcoholic fatty liver disease [30].

Most physicians (85.3%) reported using DPP-4is, either alone or in combination with other antidiabetic medications. Regarding contraindications, 89% of physicians were aware of pregnancy and breastfeeding contraindications but only 77.5% knew to discontinue DPP-4is 90 days before conception. This gap indicates a need for focused education on preconception and prenatal medication management. DPP4is are excellent candidates due to their minimal risk of hypoglycemia or weight gain, lack of need for dose escalation, and favorable safety profile. A promising diabetes treatment would control blood sugar and overall risk factors [31]. A 2018 internal and FM physician survey revealed significant knowledge gaps about cardiovascular outcomes trials (CVOTs) for type 2 diabetes. Nearly 40% were unfamiliar with the ADA's guidelines for patients with cardiovascular disease, and many were unaware of key CVOT results. This suggests that gaps in knowledge about diabetes treatments, including DPP-4is, could affect their appropriate use in high-risk patients [32]. About 77% of physicians preferred DPP-4i, and among them, nearly 56% were prescribed DPP-4i along with metformin [33].

Physicians' knowledge of DPP-4i was significantly higher among FM specialists and consultants (95.2%) than among GPs and FM residents (77.6%) (p=0.001). A similar trend was observed for SGLT-2is, with specialists demonstrating greater awareness (97.6%) than residents (80.4%) (p=0.0001). Moreover, daily patient encounters correlated with higher knowledge levels for both DPP-4is and SGLT-2is. These results suggest that both clinical experience and frequent patient interactions enhance physicians' knowledge.

Although 88.5% of physicians were aware of contraindications such as pregnancy and breastfeeding, only 78% knew to discontinue SGLT-2is 90 days before conception, and 75.9% were aware of discontinuation protocols before elective surgery. These gaps highlight a need for enhanced training on preoperative and prenatal management with SGLT-2is.

The National Institute for Health and Care Excellence guideline recommends SGLT-2is with metformin as a first-line T2DM treatment. Primary care providers should recognize the benefits of SGLT-2i and consider cardiorenal metabolic patients' prescription of these drugs [34,35].

In the present study, physicians' knowledge of SGLT-2i doses did not show a significant difference across age groups (p=0.173), and knowledge levels were similar between male (87.4%) and female physicians (88.9%) (p=0.759). However, a significant difference was observed in relation to professional position, with FM specialists and consultants demonstrating a higher level of awareness (97.6%) than GPs and FM residents (80.4%) (p=0.0001). Additionally, the frequency of patient encounters played a key role in influencing knowledge levels. Physicians who saw patients daily exhibited a significantly higher level of knowledge (90.5%) compared to those who saw patients every two days (70.6%) or weekly (66.7%). These findings emphasize that professional position and patient encounter frequency significantly influenced physicians' knowledge of DPP-4is and SGLT-2is, aligning with the insights shared in studies that underscore the importance of clinical experience and frequent exposure in improving knowledge of newer diabetes medications [32-35].

One of the study's major strengths was that it included all three novel diabetes medications among PHCC physicians, who interact as frontline caretakers. A good sample (n=191) represented wide coverage across multiple governorates in Qassim province, which enhanced the generalizability of the findings, and all physicians were included in the selected governorates. A few limitations of the study were that the online self-administered questionnaire may have led to a misunderstanding of some questions and introduced self-reporting bias, as the physical presence of the principal investigator was lacking while filling out the questionnaire. PHCC physicians who regularly manage T2DM make the results highly relevant to current real-world practice. Additionally, it highlights the current level of awareness regarding newer classes of antidiabetic medication.

## Conclusions

The study concluded that awareness about diabetes medications (GLP-1 RA, DPP-4i, and SGLT-2i) among Qassim PHCC physicians is good. Close to one-third (36.1%) of the physicians received CME in spite of daily encounters with patients with T2DM in their clinics. Most physicians (92.7%) acknowledged that GLP-1 RA medication is superior in HbA1c reduction and cardiovascular benefits. Policymakers should focus on CME programs, updated guidelines, and tailored training to close knowledge-practice gaps.

Based on the study findings, we recommend that policymakers should prioritize structured CME programs focusing on novel diabetes medications, particularly GLP-1 RAs. These programs should target general practitioners and FM residents with the most significant knowledge gaps. Additionally, once GLP-1 RA becomes available in PHCCs, future researchers are encouraged to assess how physicians integrate these medication prescriptions into their daily practice in order to evaluate the current application and treatment outcomes.

## Appendices

Questionnaire: 

Demographic Characteristics: 

1. Age of the physician: ------------(Completed years)

2. Gender of the family physician: 1. Male 2. Female

3. Years of experience as a physician: ------------(Completed years)

4. Position of the doctor: 1. General Physician 2. FM Resident 3. FM Specialist 4. FM Consultant

5. Frequency of encounters of patients with type 2 diabetes: 1. Daily 2. Once in 2 days 3. Weekly once.

6. Did you receive any CME on a novel class of drugs management of type 2 diabetes for the last year? 1. No 2. Yes

7. Availability of educational resources on GLP-1 receptor agonists? 1. No 2. Yes

8. Availability of educational resources on DPP-4 inhibitors? 1. No 2. Yes

9. Availability of educational resources on SGLT-2 inhibitors? 1. No 2. Yes

10. Which time do you prefer to use the GLP-1 class of drugs in your practice? 1. First-line drugs fail for T2DM control 2. Any time use irrespective of control status of T2DM.

11. Which time do you prefer to use the DPP-4 class of drugs in your practice? 1. First-line drugs fail for T2DM control 2. Any time use irrespective of control status of T2DM.

12. Which time do you prefer to use the SGLT-2 receptor class of drugs in your practice? 1. First-line drugs fail for T2DM control 2. Any time use irrespective of control status of T2DM.

13. In your experience how much reduction of HbA1C with 6 months of treatment with GLP-1 class of drugs? ----------- %

14. In your experience how much reduction of HbA1C with 6 months of treatment with DPP-4 class of drugs? ----------- %

15. In your experience how much reduction of HbA1C was reduced with 6 months of treatment with the SGLT-2 receptor class of drugs? ----------- %

16. Awareness of the latest clinical ADA guidelines for the use of GLP-1 receptor agonists? 1. No 2. Yes

17. Awareness of the latest clinical ADA guidelines for the use of the DPP-4 class of drugs? 1. No 2. Yes

18. Awareness of the latest clinical ADA guidelines for the use of the SGLT-2 receptor class of drugs? 1. No 2. Yes

**Table 10:** Knowledge of GLP-1 RA

Sl. No	Knowledge questions about GLP-1 RA	Yes	No
1.	Do you know glucagon-like peptide-1 receptor agonists (GLP-1 RA)?		
2.	Do you know that GLP-1 RA is the latest addition in the management of type-2 diabetes mellitus?		
3.	Is it true that administration of GLP-1 RA does not cause hypoglycemia in diabetes type-II patients?		
4.	Is it true that the use of GLP-1 RA can cause a reduction in blood pressure?		
5.	Are you aware that GLP-1 RA therapy can cause pancreatitis in diabetes patients?		
6.	Do you agree that GLP-1 RA carries a potential risk of thyroid C-cell neoplasm?		
7.	Are you aware of ADA recommendations in 2024 in regard to GLP-1?		
8.	Do you agree that severe renal impairment is considered a contraindication for GLP?		
9.	Do you agree that pregnancy and breastfeeding are considered as a contraindication for GLP?		
10.	Do you agree that gastroparesis is considered a contraindication for GLP?		
11.	Are aware of different doses of GLP?		
12.	Are you aware of GLP1 drug routes of administration?		
13.	Do you understand the role of GLP-1 RA in reducing cardiovascular risk factors in patients with diabetes?		
	Knowledge Questions about DPP-4 Inhibitors		
14.	Are you aware of different doses of DPP-4 inhibitors?		
15.	Are you aware of the route of administration of DDP-4 inhibitors?		
16.	Do you use DPP-4 inhibitors alone or in combination with other novel classes of diabetic medication (SGLT-2 inhibitors)		
17.	Do you agree that pregnancy and breastfeeding are considered contraindications for DPP-4?		
18.	Do you agree that 90 days before conception of pregnancy, the DPP-4 inhibitors must be stopped?		
20.	Are you aware of the route of administration of SGLT-2 inhibitors?		
21.	Do you agree that pregnancy and breastfeeding are considered contraindications for SGLT-2?		
22.	Do you agree that 90 days before conception of pregnancy, the SGLT-2 inhibitors must be stopped?		
23.	Do you know SGLT-2 inhibitors are to be stopped 3 days before elective surgery?		

**Table 11:** Knowledge of SGLT-2i and DPP-4i

16.	Do you use DPP-4 inhibitors alone or in combination with other novel classes of diabetic medication (SGLT-2 inhibitors)		
17.	Do you agree that pregnancy and breastfeeding are considered contraindications for DPP-4?		
18.	Do you agree that 90 days before conception of pregnancy, the DPP-4 inhibitors must be stopped?		
	Knowledge questions about SGLT-2 inhibitors		
19.	Are you aware of different doses of SGLT-2 inhibitors?		
20.	Are you aware of the route of administration of SGLT-2 inhibitors?		
21.	Do you agree that pregnancy and breastfeeding are considered contraindications for SGLT-2?		
22.	Do you agree that 90 days before conception of pregnancy, the SGLT-2 inhibitors must be stopped?		
23.	Do you know SGLT-2 inhibitors are to be stopped 3 days before elective surgery?		

## References

[1] Antar SA, Ashour NA, Sharaky M (2023). Diabetes mellitus: classification, mediators, and complications; a gate to identify potential targets for the development of new effective treatments. Biomed Pharmacother.

[2] Wang H, Li N, Chivese T (2022). IDF Diabetes Atlas: estimation of global and regional gestational diabetes mellitus prevalence for 2021 by International Association of Diabetes in Pregnancy Study Group's criteria. Diabetes Res Clin Pract.

[3] Alzughbi T, Badedi M, Darraj H, Hummadi A, Jaddoh S, Solan Y, Sabai A (2020). Diabetes-related distress and depression in Saudis with type 2 diabetes. Psychol Res Behav Manag.

[4] Al Dawish MA, Robert AA, Braham R, Al Hayek AA, Al Saeed A, Ahmed RA, Al Sabaan FS (2016). Diabetes mellitus in Saudi Arabia: a review of the recent literature. Curr Diabetes Rev.

[5] Zheng Y, Ley SH, Hu FB (2018). Global aetiology and epidemiology of type 2 diabetes mellitus and its complications. Nat Rev Endocrinol.

[6] Tan SY, Mei Wong JL, Sim YJ (2019). Type 1 and 2 diabetes mellitus: a review on current treatment approach and gene therapy as potential intervention. Diabetes Metab Syndr.

[7] Kostial C, Manuwald U, Schulze J, Kugler J, Rothe U (2020). Disease-management-programs in the field of diabetes mellitus with identification of the best practice in Europe: a scoping review. Horm Metab Res.

[8] Zhou Z, Sun B, Huang S, Jia W, Yu D (2019). The tRNA-associated dysregulation in diabetes mellitus. Metabolism.

[9] Mabilleau G, Gobron B, Bouvard B, Chappard D (2018). Incretin-based therapy for the treatment of bone fragility in diabetes mellitus. Peptides.

[10] Nauck MA, Quast DR, Wefers J, Meier JJ (2021). GLP-1 receptor agonists in the treatment of type 2 diabetes - state-of-the-art. Mol Metab.

[11] Kristensen SL, Rørth R, Jhund PS (2019). Cardiovascular, mortality, and kidney outcomes with GLP-1 receptor agonists in patients with type 2 diabetes: a systematic review and meta-analysis of cardiovascular outcome trials. Lancet Diabetes Endocrinol.

[12] Solanki JD, Sheth NS, Shah CJ, Mehta HB (2017). Knowledge, attitude, and practice of urban Gujarati type 2 diabetics: prevalence and impact on disease control. J Educ Health Promot.

[13] Das Choudhury S, Das SK, Hazra A (2014). Survey of knowledge-attitude-practice concerning insulin use in adult diabetic patients in eastern India. Indian J Pharmacol.

[14] Mehralian G, Yousefi N, Hashemian F, Maleksabet H (2014). Knowledge, attitude and practice of pharmacists regarding dietary supplements: a community pharmacy-based survey in Tehran. Iran J Pharm Res.

[15] Widyahening IS, Khunti K, Vos RC, Chew BH (2022). Editorial: achieving effective management and treatment of diabetes mellitus in future primary care. Front Endocrinol (Lausanne).

[16] Hamed K, Alosaimi MN, Ali BA (2024). Glucagon-like peptide-1 (GLP-1) receptor agonists: exploring their impact on diabetes, obesity, and cardiovascular health through a comprehensive literature review. Cureus.

[17] Matza LS, Curtis SE, Jordan JB, Adetunji O, Martin SA, Boye KS (2016). Physician perceptions of GLP-1 receptor agonists in the UK. Curr Med Res Opin.

[18] Beaser RS, Okeke E, Neighbours J, Brown J, Ronk K, Wolyniec WW (2011). Coordinated primary and specialty care for type 2 diabetes mellitus, guidelines, and systems: an educational needs assessment. Endocr Pract.

[19] Aldhobaib AY, Rabbani SI, Mobark MA. (2021). Knowledge, attitude and practice about a newer class of antidiabetic drug (glucagon-like peptide-1 receptor agonist) among the health care professionals of Qassim University, Saudi Arabia. J Pharm Res.

[20] Korayem GB, Alshaya OA, Alghamdi AA (2022). The prescribing pattern of sodium-glucose cotransporter-2 inhibitors and glucagon-like peptide-1 receptor agonists in patient with type two diabetes mellitus: a two-center retrospective cross-sectional study. Front Public Health.

[21] Wikipedia. Al-Qassim Province April 2025 (2025). Wikipedia: Al-Qassim Province. https://en.wikipedia.org/wiki/Al-Qassim_Province.

[22] Dean AG SK, Soe MM (2025). Al-Qassim Province. Open Source Epidemiologic Statistics for Public Health 6th April.

[23] Health Mo (2025). OpenEpi: open source epidemiologic statistics for public health. Statistical year book.

[24] Inoue H, Tamaki Y, Kashihara Y, Muraki S, Kakara M, Hirota T, Ieiri I (2019). Efficacy of DPP-4 inhibitors, GLP-1 analogues, and SGLT2 inhibitors as add-ons to metformin monotherapy in T2DM patients: a model-based meta-analysis. Br J Clin Pharmacol.

[25] Ma H, Lin YH, Dai LZ, Lin CS, Huang Y, Liu SY (2023). Efficacy and safety of GLP-1 receptor agonists versus SGLT-2 inhibitors in overweight/obese patients with or without diabetes mellitus: a systematic review and network meta-analysis. BMJ Open.

[26] Chai S, Niu Y, Liu F, Wu S, Yang Z, Sun F (2024). Comparison of GLP-1 receptor agonists, SGLT-2 inhibitors, and DPP-4 inhibitors as an add-on drug to insulin combined with oral hypoglycemic drugs: umbrella review. J Diabetes Res.

[27] Gilbert MP, Pratley RE (2020). GLP-1 analogs and DPP-4 inhibitors in type 2 diabetes therapy: review of head-to-head clinical trials. Front Endocrinol (Lausanne).

[28] Ayoub M, Chela H, Amin N, Hunter R, Anwar J, Tahan V, Daglilar E (2025). Pancreatitis risk associated with GLP-1 receptor agonists, considered as a single class, in a comorbidity-free subgroup of type 2 diabetes patients in the United States: a propensity score-matched analysis. J Clin Med.

[29] Egan AG, Blind E, Dunder K, de Graeff PA, Hummer BT, Bourcier T, Rosebraugh C (2014). Pancreatic safety of incretin-based drugs--FDA and EMA assessment. N Engl J Med.

[30] Garvey WT, Mahle CD, Bell T, Kushner RF (2024). Healthcare professionals' perceptions and management of obesity & knowledge of glucagon, GLP-1, GIP receptor agonists, and dual agonists. Obes Sci Pract.

[31] Saini K, Sharma S, Khan Y (2023). DPP-4 inhibitors for treating T2DM - hype or hope? an analysis based on the current literature. Front Mol Biosci.

[32] Shubrook JH, Pak J, Dailey G (2020). Primary care physicians' knowledge of the cardiovascular effects of diabetes medications: findings from an online survey. Adv Ther.

[33] Manjula S, Krishna Kumar M (2024). Clinicians perspectives on the utilization of antidiabetics for the management of diabetes in India. World J Adv Res Rev.

[34] Evans M, Morgan AR, Bain SC (2022). Defining the role of SGLT2 inhibitors in primary care: time to think differently. Diabetes Ther.

[35] (2025). Type 2 diabetes in adults: management. https://www.nice.org.uk/guidance/ng28.

